# 

**Published:** 1846-04

**Authors:** 


### BIBLIOGRAPHICAL RECORD.

1. The Retrospect of Practical Medicine and Surgery; being a half-yearly Journal, containing a retrospective view of every discovery and practical improvement in the medical sciences. Edited by *W Braithwaite.* Vol. 12. July—December, 1845. 12mo, pp. 414. London, 1846.

2. Lectures on the Nature and Treatment of Deformities, delivered at the Royal Orthopædic Hospital, by *R. W. Tamplin*, F.R.C.S.E. 12mo, pp 279. London, 1846.

3. Observations and Essays on the Statistics of Insanity, including an enquiry into the causes influencing the results of treatment in Establishments for the Insane. To which are added the statistics of the Retreat near York. By *John Thurnam.* 8vo. 1845.

*In our next,*

4. The Half-yearly Abstract of the Medical Sciences; being a practical and analytical digest of the contents of the principal British and Continental Medical Works published in the preceding six months; together with a series of critical reports on the progress of medicine and the collateral sciences during the same period. Edited by *W. H. Ranking*, M.D. Vol. II. 8vo, pp. 472. London, 1845.

5. Monthly Journal of Medical Science. Edited by *John Rose Cormack*, M.D. January, February, and March, 1846.

6. A System of Practical Surgery. By *William Fergusson*, F.R.S.E. Second edition, 12mo, pp. 683. London, 1846.
*The best manual of Practical Surgery in onr language.*

7. Dr. Quain's Anatomy. Fifth edition, edited by Mr. *Quain* and Dr. *Sharpey.* Part II. London, 1846.

8. An Essay on the Treatment of Compound and Complicated Fractures; being the Annual Address before the Massachussetts' Medical Society in Boston, U. S. America. May 28th, 1845. By *William T. Walker*, M.D. 8vo, pp. 56. Boston, 1846. *In our next.*

9. Mercury in Fevers, Dysentery, and Hepa titis, as they occur in India, and with reference to Lesions in Mucous Surfaces and Glandular Surfaces. By *John Stuart Sutherland*, M.D. 8vo, pp. 95. Edinburgh, 1846.
*The production of an observing and thoughtful practitioner. Want of space alone prevents our noticing it at greater length.*

10. The Practice of Surgery. By *James Miller*, F.R.S.E., &c. Small 8vo, pp. 711. Edinburgh, 1846.
*A pendant to the author's "Principles:" it will prove a useful text-book to students.*

11. Transactions of the Provincial Medical and Surgical Association. New Series. Vol, II. 8vo, pp. 285. London, 1846.

12. Beitrage zur Ohrenheilkunde. Von Dr. *Wilhelm Kramer.* 8vo, pp. 314. Berlin, 1845.

*In our next.*

13. Tubercle of the Brain in Children. By *James Maxwell Adams.* 8vo. pp. 32. Glasgow, 1846.

14. The Medical Examiner and Record of Medical Science. Edited by *Robert M. Huston*, M.D. Nos. 11 and12. Philadelphia, 1845-6.

15. Abstract of Researches on Magnetism, and on certain allied subjects, including a supposed new imponderable. By Baron *Von Reichenbach.* Translated and abridged from the German, by *William Gregory*, M.D. 8vo, pp. 122. London, 1846.

16. Lectures illustrative of various Subjects in Pathology and Surgery. By Sir *Benjamin C. Brodie*, Bart. 8vo, pp. 418. London, 1846.

*In our next.*

17. A Manual of Medical Jurisprudence. By *Alfred S. Taylor.* Second edition, 8vo. pp. 720. London, 1846.

*An admirable work.*

18. Transactions of the Medical Society of London. New Series. Vol. I. With plates. 8vo, pp. London, 1846.

19. The American Journal of Insanity. Edited by the Officers of the New York State Lunatic Asylum. October, 1845.

20. The New Orleans Medical and Surgical Journal, devoted to Medicine and the Collateral Sciences. Jan. 1846.

21. A Practical Treatise on Abdominal Hernia. By *Thomas Pridgin Teale*, F.L.S. With numerous illustrations. 8vo, pp. 398. London, 1846.

22. The Cyclopædia of Anatomy and Physiology. Edited by *R. B. Todd*, M.D., &c. Part 27.

23. Medical Notes on China. By *John Wilson*, M.D. 8vo, pp. 286. London.

*In our next.*

24. Tavernier's Treatise on the Treatment of Deformities of the Spine by the "Lever-belt with Inclination Busk," &c. Translated by *W. Brewer*, M.D. 8vo, pp. 43. 1846.

*This translation is not well done; nor can the exclusive recommendation of any one apparatus be approved of.*

25. An Account of the Anatomy of the Trichocephalus Affinis. By *Erasmus Wilson*, F.R.S., &c. Pp. 12, with a plate.

*Well drawn-up and cleverly illustrated.*

26. A Statistical Nosology, for the use of those who return the Causes of Death, under 6 and 7 Will. IV. New edition. From the Registrar-General.
*We earnestly recommend medical men to attend to the "Suggestions" at p. 12, 13.*

27. Phrenology—its Nature and Uses. An Address to the Students of Anderson's University, at the opening of Dr. Weir's first course of Lectures on Phrenology in that Institution. By *Andrew Combe*, M.D. &c. 8vo, pp. 32. Edinburgh, 1846.
*An eloquent and stirring appeal, worthy of general perusal.*

28. The Application of Prismatic Reflection to the Investigation of Disease in the open Cavities of the Body. By *A. Warden*, M.D. 8vo, pp. 12, with two plates.
*Ingenious and likely to be useful.*

